# The impact paradox: mixed-methods evaluation of National Institute of Health and Care Research funding for intellectual disability research in the UK

**DOI:** 10.1192/bjo.2026.11023

**Published:** 2026-04-21

**Authors:** Nathan Goddard, Madeleine Dale, Ruth Bishop, Samuel Tromans, Nathan Johnson, Maxine Hough, Sarah Lennard, Richard A. Laugharne, Rohit Shankar

**Affiliations:** Royal Cornwall Hospitals NHS Trust, Truro, UK; University Hospitals Plymouth NHS Trust, Plymouth, UK; Cornwall Intellectual Disability Equitable Research (CIDER), Cornwall Partnership NHS Foundation Trust, Truro, UK; Peninsula School of Medicine, University of Plymouth, Truro, UK; Division of Public Health and Epidemiology, University of Leicester, Leicester, UK; Adult Learning Disability Service, Leicestershire Partnership NHS Trust, Leicester, UK; National Institute for Health and Care Research (NIHR) Clinical Research Network South West Peninsula, Exeter, UK

**Keywords:** Clinical trials, intellectual disability, neurodevelopmental disorders, randomised controlled trial, evidence-based mental health

## Abstract

**Background:**

People with intellectual disability experience substantial health inequities, including higher multimorbidity, increased healthcare utilisation and markedly reduced life expectancy. High-quality research is essential to address these disparities. The National Institute for Health and Care Research (NIHR) funded Research Delivery Network provides the infrastructure/expertise/support needed to deliver NIHR-funded studies, and supports studies funded by a non-commercial/industry partner. However, the effectiveness of NIHR-funded studies versus those supported in driving impactful intellectual disability research remains unclear.

**Aims:**

To evaluate and compare the outcomes of NIHR-funded and supported intellectual disability research.

**Method:**

All NIHR studies (funded/supported) relating to intellectual disability (2010–2020) were identified through systematic register searches. Primary outcomes included publication rates and impact on local, national and international clinical guidelines. Data collection was supplemented with a questionnaire to chief investigators and literature searches. Quantitative analyses examined associations between funding status, study design, publication and guideline impact, whereas qualitative responses explored implementation challenges.

**Results:**

In total, 88 projects were identified, and 42% (37/88) were NIHR-funded. Overall, 81% of studies generated at least one publication and 28% informed clinical guidelines. NIHR funding was not significantly associated with publication or guideline impact. Randomised controlled trials (RCTs) were significantly more likely to be published and more likely to influence non-UK national and international guidelines than non-RCTs. The amount of funding showed no association with impact. Qualitative findings highlighted funding constraints, staff capacity and stakeholder engagement as key determinants of implementation.

**Conclusions:**

NIHR-funded intellectual disability research was no more likely than NIHR-supported studies to result in publications or guideline impact.

Since its inception in 2006, the National Institute of Health and Care Research (NIHR) in the UK has aimed to fund health, public health and social care research that will lead to improved outcomes for patients and the public.^1^ The NIHR states, under its operating principles, that they aim to improve the efficacy of research and to generate evidence that enables more effective clinical care.^2^ To achieve this, it is currently financed with £1 billion annually from the Department of Health and Social Care.^3^ Although there have been articles commissioned by the Department of Health,^3^ independent studies examining the efficacy of NIHR funding and the value of medical research remain limited.^4^

## Intellectual disability

Intellectual disabilities (also known as learning disabilities in the UK) are disorders of intellectual development as defined in the ICD-11. They are characterised by significant deficits in cognitive and adaptive functioning, with onset during the developmental period.^5^ Intellectual disability affects approximately 1% of the global population, presenting unique challenges for healthcare systems.^6^ It has been shown that people with intellectual disability have poorer health outcomes than the general population, with increased rates of multimorbidity, polypharmacy and chronic health complications such as diabetes.^7,8^ People with intellectual disability are also almost twice as likely to suffer an avoidable death compared with the general population (38.8 *v*. 21.6%).^9^ These poorer health outcomes lead to a disproportionate burden on healthcare services by causing higher rates of admission.^7^ The disparity in health outcomes is highlighted by morbidity rates: the median age at death in 2023 for males and females with an intellectual disability living in England was 62.5 years compared with 82.0 years for the general population.^9^

To address these disparities in health outcomes, research focused on people with intellectual disability is crucial, and yet there remains a recognised sparsity.^10^ Long-standing barriers, including ethical concerns, communication difficulties and logistical challenges, have limited the participation of people with intellectual disability in research.^11,12^ Increased high-quality and clinically relevant research involving people with intellectual disability could lead not only to health benefits for this group, but also reduced pressure on the wider healthcare system.^10^

## Has the NIHR funded meaningful research for people with intellectual disability?

Evaluating the impact of NIHR funding can be multifaceted, with many factors holding relevance beyond just patient outcomes.^13,14^ These evaluations often have to be conducted retrospectively because it is estimated to take an average of 17 years for new research to be implemented into clinical practice.^14,15^ As the NIHR aims for its funding to improve outcomes for patients and the public,^1^ the funded research must be eventually effectively implemented in clinical practice. Recent studies on knowledge translation continue to highlight the persistent challenges in ensuring that research findings are embedded into clinical practice, explaining the delay in clinical implementation of medical research.^16^

The aim of this study was to evaluate the impact of NIHR grants awarded between 2010 and 2020 for research involving people with intellectual disability, using a patient-centred approach. Our primary aim was to evaluate the efficacy of NIHR funding in the field of intellectual disability by exploring whether awarded grants resulted in publications in index-linked scientific journals and/or whether they had an impact on national guidance/policy. We compared this to studies that were not NIHR-funded, but were NIHR-supported and on the NIHR portfolio. NIHR portfolio studies are studies that are eligible for NIHR research delivery network support, where researchers have applied for portfolio support that was subsequently approved.^17^ This is usually granted for studies that have been funded through competitive peer-reviewed grants. The NIHR portfolio contains both NIHR-funded and NIHR-supported studies.

This was determined by establishing whether these grants led to (a) published literature in scientific journals, (b) changes to local or national guidance or (c) changes to National Institute for Health and Care Excellence (NICE) guidance or other national/international guidelines.

Our secondary aims were to explore the barriers and facilitators influencing the implementation of research findings into clinical practice, and to identify the characteristics of the studies receiving funding from the NIHR for intellectual disability research. We also compared the number of these studies to funded studies on other neurodevelopmental disorders, i.e. autism and attention-deficit hyperactivity disorder (ADHD).

## Method

To address our aims, we analysed all NIHR portfolio studies conducted between 2010 and 2020 in the field of intellectual disability, incorporating both qualitative and quantitative research. We chose this time frame because of data availability, as the NIHR funding and awards website was found to be more likely to be missing publications or, indeed, the project in its entirety with older projects. We also anticipated that it would be more challenging to contact the project leads of older projects, because of outdated contact details, project leads being more likely to have retired or not being contactable for other reasons.

### Data collection

#### NIHR register search

We identified all grants given from 2010 to 2020 in all major streams of the NIHR – Research for Patient Benefit/Health Technology Assessment programme grants and included studies adopted to the NIHR portfolio. These studies can be referred to as NIHR-supported (or NIHR non-funded) projects. To search the NIHR register, we used the following search strategy: ‘StudyPortfolioQualificationDateYrMonth’ was filtered for ≤2020 AND ≥2010 and ‘StudyTitle’ filtered to include ‘intellectual disabil’, ‘learning disabil’ or ‘developmental disabil’. Recruitment counts of >0 and =0 were both included.

Using the NIHR register, we also collected data for the total number of NIHR-funded studies between 2010 and 2020 for intellectual disabilities, ADHD and autism. This was done by filtering ‘StudyPortfolioQualificationDateYrMonth’ for ≤2020 AND ≥2010 and ‘StudyFunders_Concatenated’ filtered for ‘NIHR’. Then the ‘StudyTitle’ was filtered for ‘Intellectual disabil’, ‘learning disabil’ or ‘developmental disabil’ for intellectual disability studies, ‘ADHD’ for ADHD studies and ‘Autism’, ‘Autistic’ or ‘Asperg’ for autism studies. Please note that the purpose of collecting data relating to autism and ADHD was for the purpose of comparing the number of NIHR-funded studies with that for intellectual disability, and these studies were not included within the data-set.

We also collected data for the total number of all NIHR-funded studies over the time period 2010 to 2020 and for each individual year. We did this by filtering ‘StudyFunders_Concatenated’ for ‘NIHR’ and then filtering ‘StudyPortfolioQualificationDateYrMonth’ for ≤2020 AND ≥2010, and then filtering for each individual year by =2010, =2011, etc.

#### Questionnaire

We sent a short questionnaire to all of the chief investigator email addresses identified from our search (see Supplementary Table 1). The chief investigators were given 2 weeks to respond, after which they were sent a follow-up email. If a project lead provided a partial response or indicated they would reply at a later date, they received one additional follow-up email.

#### Literature search

For all projects where we did not receive a full response to our questionnaire (*n* = 66, 75%), we conducted an online search to identify published literature from these projects. To do this, we used the NIHR journals library alongside search engines such as the PubMed and Trip databases. If this yielded no results, then a full list of publications from the project lead were identified using either university search engines or the person search function on ResearchGate.^18^ All identified published articles were then reviewed and all citations were checked using Scopus and AltMetric to ascertain any impact the published literature had on local or national guidelines. We looked for impact in UK national guidelines, non-UK national guidelines (examples included Dutch or Danish national guidelines) and international guidelines (multi-national organisations such as the World Health Organization).

Additionally, we collected detailed information about each NIHR portfolio project. Published papers were used to identify primary funding sources, whereas the NIHR Funding and Awards website^19^ provided information about the funding stream and grant amount for all NIHR-funded research A further review of the published literature from each portfolio project allowed us to determine the methodology, the UK location where the project was conducted and key characteristics including whether it was observational or interventional, focused on biological, social or psychological topics, and whether the project involved key aspects such as technology, genetics or medications.

We then consolidated the responses from project leaders with the data we had collected into a single spreadsheet, anonymised all entries and conducted statistical analysis alongside a review of the qualitative data obtained from the questionnaire responses.

### Statistical analysis

Statistical analysis was conducted using the R environment for statistical computing.^20^

Fisher’s exact tests were used to test the association between: (a) NIHR funding status (received funding compared to did not receive funding) and whether the project changed guidelines; (b) NIHR funding status and whether the project resulted in at least one publication; (c) NIHR funding status and whether the project resulted in multiple (two or more) publications; (d) randomised controlled trial (RCT) status (RCT versus non-RCT) and whether the project changed guidelines; and (e) RCT status and whether the project resulted in at least one publication.

A Wilcoxon rank-sum test was used to compare whether NIHR-funding status was associated with the median number of publications per project. Wilcoxon rank-sum tests were also used to compare whether the total grant received per project was associated with (a) project publication and (b) guideline impact. Guideline impact was assessed four times: UK national guideline impact, non-UK national guideline impact, international guideline impact and any guideline impact falling under the former three categories. *Post hoc* Monte Carlo simulations using 10 000 replicates were used to estimate the statistical power of each test, based on the observed proportions in our data-set.

### Ethics and governance

Ethics committee approval was not required as there was no intervention-based research and no patient data collection. For the questionnaire sent to research leads, no participant identifier data was collected. Further, it was to a professional participant group where consent was implicit by participation. All participants were advised at the start of the study that participation was voluntary, and informed consent would be presumed if the survey was submitted. If they chose to participate, data would be pooled, anonymised and analysed. Explicit consent to use data anonymously for research and publication was obtained. This was in a written format of ticking a box to use consent to use data for publication. This study was not registered with a public trials registry. The authors assert that all procedures contributing to this work comply with the ethical standards of the relevant national and institutional committees on human experimentation and with the Helsinki Declaration of 1975, as revised in 2013.

## Results

Of 88 portfolio projects identified, 25% (22/88) projects gave full responses to our questionnaire. For the remaining 66 projects, we relied on the data collected from our literature search.

A total of 81% (71/88) of the identified projects resulted in at least one publication. Of the identified projects, 42% (37/88) had received NIHR funding; 35% (13/37, 35%) of these NIHR-funded projects were RCTs and 84% (31/37) were published. Of the 65% (24/37) of NIHR-funded projects that were non-RCTs, 75% (18/24) of these had at least one publication. Overall, 24% (21/88) of the identified projects were RCTs. All of these were published (21/21) and 62% (13/21) of these had received NIHR funding.

### Guideline change

Overall, 28% of the identified projects resulted in some guideline change (25/88). Of all identified projects, 19% (17/88) of projects specifically affected UK national guidelines, 10% (10/88) of projects affected non-UK national guidelines and 8% (7/88) of projects affected international guidelines (Table 1). It took on average 2 years and 10 months from article publication to guideline impact for these 25 projects. Ten (10/25, 40%) projects resulted in change to multiple guidelines. On average, it took a further 17 months (4 years and 3 months in total) for there to be an impact on a second guideline. Notable guidelines that the projects had an impact on were the European guideline on the ‘Assessment and Diagnosis of Psychiatric Disorders in Adults with Intellectual Disabilities’,^21^ the ‘International Guidelines for the Management of Type 2 Diabetes for Adults with Intellectual and Developmental Disabilities’^22^ and NICE guidelines on ‘Type 2 Diabetes: Prevention in People at High Risk’.^23^

**Table 1:** Projects resulting in changes to guidelines

Region	Project
UK national guidelines	BEAT-IT: A randomised controlled trial comparing a behavioural activation treatment for depression in adults with learning disabilities with an attention control
	CBT for depression or anxiety in people with mild intellectual disabilities^a^
	Confidential Inquiry into the premature deaths of people with learning disabilities^a^
	Diabetes education for adults with intellectual disabilities (ID) with Type 2 diabetes (T2D) and their family/paid carers: A pilot RCT study^a^
	Domestic violence and women with learning disabilities
	Health checks and After Health Checks Study: Annual health checks for people with learning disabilities: An exploration of experiences, follow up and self-management of health conditions^a^
	Here to Stay: People with Learning Disabilities from New Migrant Communities
	Mathematics learning disabilities from childhood to adolescence. The Premature Infants’ Skills in Mathematics2 (PRISM2) Study
	Measuring and evaluating end-of-life care in services for adults with learning disabilities in the UK
	OK-Diabetes: Managing with Learning Disability and Diabetes^a^
	Pay More Attention: Ensuring equal access to high quality hospital care and services for children and young people with and without learning disabilities: Phases 2–4
	Practice nurse health checks for adults with intellectual disabilities. Randomised controlled trial
	Promoting Access to Psychological Therapy Services for People with Learning Disabilities
	Reasonable adjustments to provide equitable assessment, screening and treatment of osteoporosis for people with learning disabilities: A feasibility study
	Screening for glucose intolerance and development of a lifestyle education programme for prevention of Type 2 diabetes in a population with learning disabilities^a^
	The impact of perceived stigma on psychological distress, treatment concordance, service use and quality of life in people with intellectual disability
	Understanding the role of DNA variation in adults with learning disability
Non-UK national guidelines	A feasibility study of a psychological intervention to address alcohol misuse for people with mild to moderate learning disabilities living in the community
	A Prospective Investigation of Possible Seizure Triggers in Adults with Epilepsy and Intellectual Disabilities
	A randomised, placebo-controlled study to investigate the efficacy and safety of Circadin® to alleviate sleep disturbances in children with neurodevelopmental disabilities^a^
	CBT for depression or anxiety in people with mild intellectual disabilities^a^
	Clinical and cost effectiveness of staff training in Positive Behaviour Support (PBS) for treating challenging behaviour in people with intellectual disability^a^
	Confidential Inquiry into the premature deaths of people with learning disabilities^a^
	Health checks and health communication with people with learning disabilities: an exploration of the use of Easy Read health information to promote health literacy during health consultations^a^
	Screening for glucose intolerance and development of a lifestyle education programme for prevention of Type 2 diabetes in a population with learning disabilities^a^
	The impact of a walking intervention of the physical activity levels and health of adults with learning disabilities
International guidelines	A randomised, placebo-controlled study to investigate the efficacy and safety of Circadin® to alleviate sleep disturbances in children with neurodevelopmental disabilities^a^
	Clinical and cost effectiveness of staff training in Positive Behaviour Support (PBS) for treating challenging behaviour in people with intellectual disability^a^
	Confidential Inquiry into the premature deaths of people with learning disabilities^a^
	Developing valid models of psychopathology experienced by adults with learning disabilities
	Diabetes education for adults with intellectual disabilities (ID) with Type 2 diabetes (T2D) and their family/paid carers: A pilot RCT study^a^
	Identifying the factors that affect the implementation of strategies to promote a safer environment for patients who have learning disabilities in NHS hospitals
	OK-Diabetes: Managing with Learning Disability and Diabetes^a^

CBT, cognitive–behavioural therapy; RCT, randomised control trial; NHS, National Health Service.

Note: All project titles are listed verbatim. The list of references for papers from these projects can be found in Supplementary information 2.

a. Contributed to changes in two categories of guidelines.

### NIHR funding and guideline impact

In total, 37 projects were NIHR-funded, and 27% of these resulted in guideline change (10/37). Fisher’s exact test found no significant association between NIHR funding and guideline impact (odds ratio 0.89, 95% CI 0.31–2.51; *p* = 1). Seventeen projects resulted in a change to UK national guidelines, of which 35% (6/17) received NIHR funding. Fisher’s exact test showed no significant association between NIHR funding and UK national guideline impact (odds ratio 0.71, 95% CI 0.19–2.37; *p* = 0.59).

Ten projects resulted in a change to non-UK national guidelines, of which 50% received NIHR funding (5/10). There was no significant association between NIHR funding and non-UK national guideline impact (odds ratio 1.43, 95% CI 0.30–6.78; *p* = 0.74). Three of the seven projects that had an impact on international guidelines received NIHR funding (odds ratio 1.04, 95% CI 0.42–6.56; *p* = 1). Full results of each Fisher’s exact test and *post hoc* power analyses can be found in Table 2.

**Table 2:** Results of Fisher’s exact tests assessing the association between NIHR-funded projects and guideline impact and publications

Impact		Count	Odds ratio	95% CI	*p*-value	*Post hoc* power analysis
Overall guideline impact						
Guideline impact	NIHR-funded	10	0.89	0.31–2.51	1	0.35
	NIHR-supported	15				
No guideline impact	NIHR-funded	27				
	NIHR-supported	36				
UK national guideline impact						
Guideline impact	NIHR-funded	6	0.71	0.19–2.37	0.59	0.33
	NIHR-supported	11				
No guideline impact	NIHR-funded	31				
	NIHR-supported	40				
Non-UK national guideline impact						
Guideline impact	NIHR-funded	5	1.43	0.30–6.78	0.74	0.07
	NIHR-supported	5				
No guideline impact	NIHR-funded	32				
	NIHR-supported	46				
International guideline impact						
Guideline impact	NIHR-funded	3	1.04	0.14–6.56	1	0.07
	NIHR-supported	4				
No guideline impact	NIHR-funded	34				
	NIHR-supported	47				
Publications						
Resulted in at least one publication	NIHR-funded	31	1.42	0.42–5.21	0.59	0.07
	NIHR-supported	40				
Did not result in a publication	NIHR-funded	6				
	NIHR-supported	11				
Multiple publications						
Resulted in two or more publications	NIHR-funded	21	1.86	0.73–4.83	0.195	0.067
	NIHR-supported	21				
Resulted in one publication	NIHR-funded	16				
	NIHR-supported	30				

NIHR, National Institute for Health and Care Research.

### NIHR funding and publication

A total of 81% of projects (71/88) resulted in at least one publication, of which 44% (31/77) were NIHR-funded. There was no significant difference between having received NIHR funding and publication (odds ratio 1.42, 95% CI 0.42–5.21; *p* = 0.59) (Table 2). Regarding the NIHR portfolio, 48% (42/88) of projects on the NIHR portfolio had multiple publications; 48% (20/42) of these were NIHR-funded and 52% (22/42) were NIHR-supported. There was no significant difference in whether a project achieved multiple publications when comparing NIHR-funded and NIHR-supported projects (odds ratio 1.86, 95% CI 0.73–4.83; *p* = 0.195). NIHR-funded projects published a total of 89 publications compared with 117 publications for all NIHR-supported projects. The median number of publications per NIHR-funded project was 2 (interquartile range (IQR) 0–4). The median number of publications per NIHR-supported project was 1 (IQR 0–2). There was no significant difference between the median number of publications from projects that were NIHR-funded compared with those that were NIHR-supported (*W* = 785; *p* = 0.17; estimated *post hoc* power: 0.29).

### RCTs and guideline impact

Of the 28% of projects (25/88) that resulted in a change to guidelines, 36% were RCTs (9/25). Fisher’s exact test found no significant association between RCT status and guideline impact (odds ratio 2.36, 95% CI 0.74–7.49; *p* = 0.104). Of the 17 projects that resulted in changes to UK national guidelines, 29% were RCTs (5/17). There was no significant association between RCT status and UK national guideline impact (odds ratio 1.43, 95% CI 0.34–5.22; *p* = 0.54). Of the ten projects that resulted in changes to non-UK national guidelines, 60% were RCTs (6/10). RCTs were more likely than non-RCTs to result in a change to guidelines in non-UK countries (odds ratio 6.13, 95% CI 1.27–33.5; *p* = 0.01). Of the seven projects that impacted international guidelines, 57% were RCTs (4/7). RCTs were more likely than non-RCTs to result in a change to international guidelines, approaching significance (odds ratio 4.90, 95% CI 0.75–36.7; *p* = 0.053). Full results of each Fisher’s exact test and *post hoc* power analyses can be found in Table 3.

**Table 3:** Results of Fisher’s exact tests assessing the association between randomised control trials and guideline impact and publications

Impact		Count	Odds ratio	95% CI	*p*-value	*Post hoc* power analysis
Overall guideline impact						
Guideline impact	RCT	9	2.36	0.74–7.49	0.104	0.20
	Non-RCT	16				
No guideline impact	RCT	12				
	Non-RCT	51				
UK national guideline impact						
Guideline impact	RCT	5	1.43	0.34–5.22	0.54	0.02
	Non-RCT	12				
No guideline impact	RCT	16				
	Non-RCT	55				
Non-UK national guideline impact						
Guideline impact	RCT	6	6.13	1.27–33.5	0.01	0.39
	Non-RCT	4				
No guideline impact	RCT	15				
	Non-RCT	63				
International guideline impact						
Guideline impact	RCT	4	4.90	0.75–36.7	0.053	0.27
	Non-RCT	3				
No guideline impact	RCT	17				
	Non-RCT	64				
Publications						
Resulted in at least one publication	RCT	21	∞	1.53–∞	0.01	0.90
	Non-RCT	50				
Did not result in a publication	RCT	0				
	Non-RCT	17				

RCT, randomised controlled trial.

### RCTs and publication

All 21 of the RCTs were published, comprising 42% (21/50) of the overall number of published projects. RCTs were significantly more likely to be published than non-RCTs (odds ratio ∞, 95% CI 1.53 to ∞; *p* = 0.009) (Table 3).

### Funding amount

Data on grant value received were available for 32 of the identified projects, all of which were NIHR-funded. We were unable to identify grant value amount for all 37 NIHR projects because the NIHR Funding and Awards website was missing this data and our NIHR analysts were not able to identify individual funding amounts. The median grant received was £346 187 (IQR £232 700 to £782 916). The minimum grant received was £19 360 and the maximum was £2 316 140. Of the projects with available grant data, five influenced UK national guidelines, five influenced non-UK national guidelines and three influenced international guidelines.

There was no significant difference in total funding between projects that resulted in a change to guidelines compared with those that did not (*W* = 83; *p* = 0.39). The estimated *post hoc* power was 0.13. In addition, there was no significant different in total funding between projects that resulted in a change to UK national guidelines (*W* = 43; *p* = 0.20; estimated *post hoc* power 0.22), a change to non-UK national guidelines (*W* = 71; *p* = 0.88; estimated *post hoc* power 0.08) or a change to international guidelines (*W* = 23; *p* = 0.20; estimated *post hoc* power 0.12), compared with those that did not. There was no significant difference in total funding between projects that were published and those that were not (*W* = 37; *p* = 0.12; estimated *post hoc* power 0.38).

### Qualitative analysis

We received 22 full responses to our questionnaire (22/88, 25%), which included responses to barriers and facilitators to the implementation of research findings into clinical practice. Although four project leads reported no barriers (4/22, 18.2%), most encountered a range of barriers. One of the most common barriers to implementation of practice was a lack of further funding identified by both NIHR-funded and NIHR-supported studies. As these are direct quotes, we are unable to delineate whether this refers to a lack of NHS funding or further grant funding. The engagement of professionals, the capacity of staff and the COVID-19 pandemic were also identified as common barriers. Examples of these barriers can be seen in Table 4.

**Table 4:** Barriers to implementation of research findings in clinical practice

A lack of further funding
‘Lack of sufficient and specific funding’
‘Lack of funding after the end of the project for impact related activities’
‘The competitive environment for securing follow-one funding’
The engagement of professionals
‘Engagement and involvement of key professionals’
‘Attitudes/culture of mental health professionals’
‘Our findings have been well received in health and social care, but little evidence that the police have heard and understood’
The capacity of staff
‘Capacity and knowledge of primary care staff to undertake the training’
‘Lack of sufficient and specific staff time to do this’
‘It was simply not possible for support workers, despite their willingness, to deliver the intervention with fidelity’
COVID-19 pandemic
‘A pandemic! I had not long collected all my data when the first lockdown occurred’
‘Then COVID happened, and they went into “survival mode” and felt that doing basic care was all they were able to do’

Four different project leads also reported there to be no facilitators (4/22, 18.2%) for the implementation of clinical research findings into clinical practice. From those who did report facilitators, the most common topics were patient engagement and having close ties to organisations. Other facilitators mentioned often revolved around further funding, either from government or other sources. Examples of these facilitators can be seen in Table 5.

**Table 5:** Facilitators to implementation of research findings in clinical practice

Patient engagement
‘Good uptake from service users once offered and high attendance at sessions’
‘Staff and participant motivation’
‘A keen interest in the topic from people with intellectual disabilities’
Close ties with organisations
‘We collaborated with Diabetes UK and continue to do so. This collaboration helped us reach many more people than we could have as a University based project’
‘Wales is a small nation and closer ties with Improvement Cymru etc. mean that you can have more influence’
‘The product was launched in the UK by our marketing partner Flynn pharma’
Further funding
‘We received small pots of money from our institution to enable us to create guidance documents based on the findings’
‘Internal university funding for impact related activities was beneficial’
Government funding
‘Department of Health funding’
‘NIHR [National Institute for Health and Care Research] funding was a facilitator to the development and conducting of the definitive RCT [randomised controlled trial]’

### NIHR funding for intellectual disability compared with other subspecialties

Between 2010 and 2020, the NIHR-funded 4503 studies. Of these 4503 studies, 37 (0.82%) were into the field of intellectual disability. This is a similar total number of NIHR-funded studies when compared to autism studies (*n* = 30) and ADHD (*n* = 16). The total number of NIHR-funded studies was trending upward until 2019, and returned to its lowest level in 2020.

## Discussion

Studies for intellectual disability funded by the NIHR between 2010 and 2020 were no more likely to be published than any other project adopted to the NIHR portfolio, which had been funded by other sources, such as charity funding. Similarly, they were not more likely to impact clinical or national guidelines. Although 35% of projects that led to changes in guidelines were RCTs, there was no significant association between RCT status and impact on UK guidelines. However, RCTs were more likely than non-RCTs to have an impact on non-UK national guidelines and international guidelines. RCTs were also significantly more likely to be published than non RCTs. There was no significant association found between funding amount and impact on national guidance.

Results do not suggest that NIHR funding does not lead to any changes to local, national or international guidelines, but rather, that studies directly funded by NIHR between 2010 and 2020 were no more likely than those studies adopted by the NIHR portfolio to have an impact on guidelines. This finding does not directly contradict the long-standing aim of the NIHR ‘to fund health, public health and social care research that will lead to improved outcomes for patients and the public’.^1,24^ However, it does indicate that receiving NIHR funding does not necessarily make a project more likely to influence clinical practice compared with those that receive funding from alternative sources.

RCTs were more likely to be published and have an impact on non-UK national guidelines and international guidelines. As RCTs are often viewed as a gold standard of research, it is not surprising that these are more likely to be published. It is possible that the highly controlled nature of these projects with their clearcut findings, makes their results more transferable to different populations and social settings globally. Therefore, it is not surprising that RCTs are statistically more likely to have an impact on non-UK national and international guidelines than non-RCTs.

With 1% of the general population estimated to have intellectual disability,^6^ 0.82% of NIHR-funded projects from 2010 to 2020 were in the field of intellectual disability. This becomes of further relevance when it is considered that patients with intellectual disability have significantly greater levels of healthcare service utilisation.^7^ However, the number of projects was similar to that in subspecialties such as autism and ADHD, suggesting a broader underfunding of research across neurodevelopmental conditions.

Further funding to research projects was perceived as crucial for the successful implementation of research into clinical practice, with access to it cited as both a facilitator and barrier. This perception persists despite our results that indicated that the amount of NIHR funding was not significantly associated with the likelihood of having an impact on national guidelines either in the UK or abroad. This view may stem from anecdotal experiences with implementation of findings into local practice.

One notable theme from the questionnaire responses was that, although people with intellectual disability were likely to be facilitators for the implementation of findings into clinical practice, clinical staff were sometimes perceived to be a barrier to the implementation of research findings. Although evidence-based medicine does encourage a level of scepticism, perhaps we could partly explain staff being a barrier through the hesitancy to work outside of guidelines, which, of course, research projects cannot affect without being trialled first. Other potential explanations for why clinical staff may be barriers to implementation could include minimal perceived benefit for patient care, a lack of awareness of the research findings or a shortage of time or resources to implement new findings.

A potential implication from our findings for the NIHR would be that perhaps they could identify common characteristics of studies that have a clinical impact such as the studies found to have impact in our study. They could then prioritise funding that shares similar characteristics, on the basis that these are more likely to tangibly affect patient care.

We based our qualitative analysis of the barriers and facilitators to implementing research findings into clinical practice on responses from project leads who completed the questionnaire. It is possible that those respondents were not fully representative of the wider pool, as they may be more likely to have had impactful studies or positive experiences with the implementation of their research findings into clinical practice. It should be noted that although we had 22 responses to our questionnaire, for the other 66 projects, we were reliant on our own online search to reveal any published literature. We then had to review the citations of all identified articles to try find any impact on guidelines. This makes it possible that there were projects that had impact on guidelines but which we were unable to identify. However, this is likely to apply for both NIHR-funded and non-NIHR-funded projects.

A key advantage of NIHR-funded projects is that the NIHR journals library makes it much easier to identify related publications. This also improves accessibility for the general public. Since the NIHR is funded by the taxpayer, it is reasonable that those who contribute financially should have the opportunity to see the impact of their taxes on new research and clinical practice.

### Strengths and limitations

*Post hoc* power analyses for several of our Fisher’s exact tests and Mann–Whitney *U*-tests revealed power <0.3, reflecting small counts and the finite size of our study: our analysis had a census nature, collating existing projects on intellectual disability. This also meant that *a priori* power calculations were not feasible. Therefore, the non-significant findings should be interpreted with their wide confidence intervals and potential for type 2 error, rather than relying only on *p*-values. For the Mann–Whitney *U*-tests, use of the asymptotic *p*-value accounted for ties in the data on total grant received. We acknowledge that these findings therefore may lack generalisability.

However, although these results may lack generalisability, this study represents an important initial step in evaluating the impact of NIHR portfolio studies on intellectual disability – something that has not previously been looked at.

During our data collection, we were only able to achieve 25% response rate from project leads despite multiple emails. To mitigate this limitation and ensure a full data collection, we made every effort to research each project for evidence of impact.

A further limitation of our study is that a project could have been conducted on a clinically important topic to a high standard and not have had an impact on clinical guidelines by virtue of generating findings that supported pre-existing practice guidelines.

It has been shown that the findings of research projects can take up to 17 years to be implemented into clinical practice.^14,15^ This is vastly different to the 2 years and 10 months figure that we found for the time that NIHR portfolio projects took to have an impact on clinical guidelines. Although his discrepancy may be explained by the 17 year figure being outdated (the paper from Morris et al^15^ is 15 years old), it could also highlight a limitation of our study, in that by taking studies from 2010 to 2020, our findings may be underestimating the longer-term impact of many of these projects, particularly for more recent projects. Although they may not have had an impact on guidelines at the time of writing, it is possible that over the next 10 years, they do end up having impact.

A reflection post study is that it would have been useful to gain the feedback of the responding principal investigators to the survey on the results, to complete a learning journey. Unfortunately, this was not factored into the study design.

In conclusion, there was no statistically significant difference in publication outputs or impact on guidelines between NIHR-funded projects and NIHR-supported projects between 2010 and 2020. This does not suggest that there is no value in NIHR funding, as there are NIHR-funded projects that have gone on to have an impact on clinical practice. Without NIHR-funding, some of these projects might have struggled to secure the funding needed to complete the high-quality research required to have that impact.

Our study suggests that the funding source of projects does not significantly affect how likely it is to impact clinical practice. This needs to be considered in the current drive of the UK government to increase commercial research.^25^ Although this initiative is welcome, non-commercial studies, particularly those being supported by non-commercial partners like charities, are inadvertently coming into competition with commercial research priorities for the NIHR research delivery network resources. The new NIHR research delivery frameworks propose looking to prioritise RCTs and/or commercial research. This could adversely affect studies for people with intellectual disability, where there is limited commercial funding and they are less likely to employ an RCT design. This policy change mirrors the lack of bespoke inclusion in the NHS 10-Year clinical delivery plan.^26^ To address these issues, active measures of equity need to be considered.

There is a relative dearth of research into the efficacy of NIHR funding. Future research would be useful to explore whether these findings are unique to the field of intellectual disability or whether similar findings are seen in other medical subspecialties, including in physical health disorders or psychiatric non-neurodevelopmental disorder. This was outside the scope of this research project.

## Supporting information

10.1192/bjo.2026.11023.sm001 Goddard et al. supplementary material 1 Goddard et al. supplementary material

10.1192/bjo.2026.11023.sm002 Goddard et al. supplementary material 2 Goddard et al. supplementary material

## Data Availability

The data that support the findings of this study are available from the corresponding author, R.S., upon reasonable request.

## Supplementary material

The supplementary material is available online at https://doi.org/10.1192/bjo.2026.11023
