# Supplementary material for: The impact paradox: mixed-methods evaluation of National Institute of Health and Care Research funding for intellectual disability research in the UK
Source: BJPsych Open. 2026 Apr 21;12(3):e111. doi: 10.1192/bjo.2026.11023 (PMC13107329; doi:10.1192/bjo.2026.11023)
Supplement: Goddard et al. supplementary material 1 — Goddard et al. supplementary material [file S2056472426110230sup001.docx]

Supplementary Table 1:

| **Question** | **Answer** |
| --- | --- |
| 1. Do we have your consent to use the data collected from the following questions to publish a paper/poster? | Yes/No (please delete the one **not** applicable) |
| 2.      What was the name of your project? |  |
| 3.      Has your project been completed? | Yes/No (please delete the one **not** applicable) |
| 4. Did your project receive NIHR funding? | Yes/No (please delete the one **not** applicable) |
| 5.      Have you published the project and if so, where was it published? Please provide the journal link/DOI |  |
| 6.      Did your project have any impact on any national guidance or policy? If so could you please provide a link to the output or outputs? |  |
| 7.      Would you please be able to offer some brief thoughts on what was done well with the project and what you think could have perhaps been done better? |  |
| 8.      What barriers did you encounter to implementing your study findings/evidence into practice? |  |
| 9.      What facilitators did you encounter to implementing your study findings/evidence into practice? |  |
| 10.      Did the project directly lead to a further successful funding application? If so please provide details. | Yes/ No (please delete the one **not** applicable). If so, please provide details |
