# Supplementary material for: The impact paradox: mixed-methods evaluation of National Institute of Health and Care Research funding for intellectual disability research in the UK
Source: BJPsych Open. 2026 Apr 21;12(3):e111. doi: 10.1192/bjo.2026.11023 (PMC13107329; doi:10.1192/bjo.2026.11023)
Supplement: Goddard et al. supplementary material 2 — Goddard et al. supplementary material [file S2056472426110230sup002.docx]

Supplementary information 2:Sample list of papers published

1. Jahoda A, Melville C, Cooper SA, Hastings R, Briggs A, Dagnan D, et al. BEAT-IT: Comparing a behavioural activation treatment for depression in adults with intellectual disabilities with an attention control: Study protocol for a randomised controlled trial. Trials [Internet]. 2015;16(1):1–11. Available from: http://dx.doi.org/10.1186/s13063-015-1103-5
2. Jahoda A, Hastings R, Hatton C, Cooper SA, Dagnan D, Zhang R, et al. Comparison of behavioural activation with guided self-help for treatment of depression in adults with intellectual disabilities: a randomised controlled trial. The Lancet Psychiatry [Internet]. 2017;4(12):909–19. Available from: http://dx.doi.org/10.1016/S2215-0366(17)30426-1
3. Jahoda A, Hastings R, Hatton C, Cooper SA, McMeekin N, Dagnan D, et al. Behavioural activation versus guided self-help for depression in adults with learning disabilities: The beatit RCT. Health Technol Assess (Rockv). 2018;22(53):vii–130.
4. Hassiotis A, Serfaty M, Azam K, Strydom A, Blizard R, Romeo R, et al. Manualised Individual Cognitive Behavioural Therapy for mood disorders in people with mild to moderate intellectual disability: A feasibility randomised controlled trial. J Affect Disord [Internet]. 2013;151(1):186–95. Available from: http://dx.doi.org/10.1016/j.jad.2013.05.076
5. Glover G, Williams R, Heslop P, Oyinlola J, Grey J. Mortality in people with intellectual disabilities in England. J Intellect Disabil Res. 2017;61(1):62–74.
6. Heslop P, Glover G. Mortality of People with Intellectual Disabilities in England: A Comparison of Data from Existing Sources. J Appl Res Intellect Disabil. 2015;28(5):414–22.
7. Heslop P, Blair PS, Fleming P, Hoghton M, Marriott A, Russ L. The Confidential Inquiry into premature deaths of people with intellectual disabilities in the UK: A population-based study. Lancet [Internet]. 2014;383(9920):889–95. Available from: http://dx.doi.org/10.1016/S0140-6736(13)62026-7
8. Taggart L, Truesdale M, Carey ME, Martin-Stacey L, Scott J, Bunting B, et al. Pilot feasibility study examining a structured self-management diabetes education programme, DESMOND-ID, targeting HbA1c in adults with intellectual disabilities. Diabet Med. 2018;35(1):137–46.
9. McCarthy M, Hunt S, Milne-Skillman K. ‘I Know it was Every Week, but I Can’t be Sure if it was Every Day: Domestic Violence and Women with Learning Disabilities. J Appl Res Intellect Disabil. 2017;30(2):269–82.
10. McCarthy M. ‘What kind of abuse is him spitting in my food?’: reflections on the similarities between disability hate crime, so-called ‘mate’ crime and domestic violence against women with intellectual disabilities. Disabil Soc [Internet]. 2017;32(4):595–600. Available from: http://dx.doi.org/10.1080/09687599.2017.1301854
11. McCarthy M, Bates C, Triantafyllopoulou P, Hunt S, Milne Skillman K. “Put bluntly, they are targeted by the worst creeps society has to offer”: Police and professionals’ views and actions relating to domestic violence and women with intellectual disabilities. J Appl Res Intellect Disabil. 2019;32(1):71–81.
12. McCarthy, M. (2019). ‘All I wanted was a happy life’: the struggles of women with learning disabilities to raise their children while also experiencing domestic violence. *Journal of Gender-Based Violence* 3, 1, 101-117
13. Cavanagh DE, Northway R, Todd S. Annual health checks for people with intellectual disabilities: An exploration of experiences, follow-up and self-management of health conditions. J Appl Res Intellect Disabil. 2024;37(3).
14. Sallah D and Kozlowska O,(2014) 'Here to Stay: Research into the needs, experiences and outcomes of health and social care services of people with learning disabilities from ethnic minority communities, including new migrants, living in England' Faculty of Psychiatry of Intellectual Disability eNewsletter 2014
15. Sallah D, Kozlowska O, Fisher B, Serrant L. The ‘ Here to Stay ’ Project Access to Services , Experience and Outcomes of Care for People with Intellectual Disabilities from Black and Minority Ethnic Communities , Including New Migrants Prevalence rates , health and social care needs and system resp. 2015;
16. Johnson S, Bamber D, Bountziouka V, Clayton S, Cragg L, Gilmore C, et al. Improving developmental and educational support for children born preterm: Evaluation of an e-learning resource for education professionals. BMJ Open. 2019;9(6):1–8.
17. Trickett K, et *al*., (2021) ‘No Excess of Mathematics Anxiety in Adolescents Born Very Preterm. J Dev Behav Pediatr, 1;42(3):220-226. doi:10.1097/DBP.0000000000000884
18. Spiller J, Clayton S, Cragg L, Johnson S, Simms V, Gilmore C. Higher level domain specific skills in mathematics; The relationship between algebra, geometry, executive function skills and mathematics achievement. PLoS One [Internet]. 2023;18(11 November):1–20. Available from: http://dx.doi.org/10.1371/journal.pone.0291796
19. Clayton S, Simms V, Cragg L, Gilmore C, Marlow N, Spong R, et al. Etiology of persistent mathematics difficulties from childhood to adolescence following very preterm birth. Child Neuropsychol [Internet]. 2022;28(1):82–98. Available from: https://doi.org/10.1080/09297049.2021.1955847
20. Bernal J, Hunt K, Worth R, Shearn J, Jones E, Lowe K, et al. Expecting the unexpected: measures, outcomes and dying trajectories for expected and unexpected death in adults with intellectual disabilities in social care settings in the UK. J Appl Res Intellect Disabil. 2021;34(2):594–605.
21. Todd S, Bernal J, Shearn J, Worth R, Jones E, Lowe K, et al. Last months of life of people with intellectual disabilities: A UK population-based study of death and dying in intellectual disability community services. J Appl Res Intellect Disabil. 2020;33(6):1245–58.
22. Northway R, Todd S, Hunt K, Hopes P, Morgan R, Shearn J, et al. Nursing care at end of life: a UK-based survey of the deaths of people living in care settings for people with intellectual disability. J Res Nurs. 2019;24(6):366–82.
23. Todd S, Bernal J, Worth R, Shearn J, Brearley S, McCarron M, et al. Hidden lives and deaths: the last months of life of people with intellectual disabilities living in long-term, generic care settings in the UK. J Appl Res Intellect Disabil. 2021;34(6):1489–98.
24. Todd S, Brandford S, Worth R, Shearn J, Bernal J. Place of death of people with intellectual disabilities: An exploratory study of death and dying within community disability service settings. J Intellect Disabil. 2021;25(3):296–311.
25. Hunt K, Bernal J, Worth R, Shearn J, Jarvis P, Jones E, et al. End-of-life care in intellectual disability: A retrospective cross-sectional study. BMJ Support Palliat Care. 2020;10(4):469–77.
26. House A, Bryant L, Russell AM, Hughes AW, Graham L, Walwyn R, et al. Managing with learning disability and diabetes: OK-Diabetes – A case-finding study and feasibility randomised controlled trial. Health Technol Assess (Rockv). 2018;22(26):1–328.
27. House A, Latchford G, Russell AM, Bryant L, Wright J, Graham E, et al. Development of a supported self-management intervention for adults with type 2 diabetes and a learning disability. Pilot Feasibility Stud. 2018;4(1):1–11.
28. House A, Bryant L, Russell AM, Wright-Hughes A, Graham L, Walwyn R, et al. Randomized controlled feasibility trial of supported self-management in adults with Type 2 diabetes mellitus and an intellectual disability: OK Diabetes. Diabet Med. 2018;35(6):776–88.
29. Mitchell F, Kirk A, Robertson K, Reilly JJ. Development and feasibility testing of an intervention to support active lifestyles in youths with type 1 diabetes-the ActivPals programme: A study protocol. Pilot Feasibility Stud [Internet]. 2016;2(1):1–11. Available from: http://dx.doi.org/10.1186/s40814-016-0106-7
30. Russell AM, Bryant L, House A. Identifying people with a learning disability: An advanced search for general practice. Br J Gen Pract. 2017;67(665):e842–50.
31. Bryant LD, Russell AM, Walwyn REA, Farrin AJ, Wright-Hughes A, Graham EH, et al. Characterizing adults with Type 2 diabetes mellitus and intellectual disability: outcomes of a case-finding study. Diabet Med. 2018;35(3):352–9.
32. Graham L, Wright J, Walwyn R, Russell AM, Bryant L, Farrin A, et al. Measurement of adherence in a randomised controlled trial of a complex intervention: supported self-management for adults with learning disability and type 2 diabetes. BMC Med Res Methodol [Internet]. 2016;16(1):1–11. Available from: http://dx.doi.org/10.1186/s12874-016-0236-x
33. Walwyn REA, Russell AM, Bryant LD, Farrin AJ, Wright-Hughes AM, Graham EH, et al. Supported self-management for adults with type 2 diabetes and a learning disability (OK-Diabetes): Study protocol for a randomised controlled feasibility trial. Trials [Internet]. 2015;16(1):1–11. Available from: http://dx.doi.org/10.1186/s13063-015-0832-9
34. Oulton K, Wray J, Kenten C, Russell J, Carr L, Hassiotis A, et al. Equal access to hospital care for children with learning disabilities and their families: a mixed-methods study. Heal Soc Care Deliv Res. 2022;10(13):i–141.
35. Macdonald S, Morrison J, Melville CA, Baltzer M, MacArthur L, Cooper SA. Embedding routine health checks for adults with intellectual disabilities in primary care: practice nurse perceptions. J Intellect Disabil Res. 2018;62(4):349–57.
36. Cooper SA, Morrison J, Allan LM, McConnachie A, Greenlaw N, Melville CA, et al. Practice nurse health checks for adults with intellectual disabilities: A cluster-design, randomised controlled trial. The Lancet Psychiatry [Internet]. 2014;1(7):511–21. Available from: http://dx.doi.org/10.1016/S2215-0366(14)00078-9
37. Chinn D, Abraham E. Using “candidacy” as a framework for understanding access to mainstream psychological treatment for people with intellectual disabilities and common mental health problems within the English Improving Access to Psychological Therapies service. J Intellect Disabil Res. 2016;60(6):571–82.
38. Finlayson J, De Amicis L, Gallacher S, Munro R, Crockett J, Godwin J, et al. Reasonable adjustments to provide equitable and inclusive assessment, screening and treatment of osteoporosis for adults with intellectual disabilities: A feasibility study. J Appl Res Intellect Disabil. 2019;32(2):300–12.
39. Dunkley AJ, Tyrer F, Spong R, Gray LJ, Gillett M, Doherty Y, et al. Screening for glucose intolerance and development of a lifestyle education programme for prevention of type 2 diabetes in a population with intellectual disabilities: the STOP Diabetes research project. Programme Grants Appl Res 2017;5(11)
40. Ali A, King M, Strydom A, Hassiotis A. Self-reported stigma and its association with socio-demographic factors and physical disability in people with intellectual disabilities: results from a cross-sectional study in England. Soc Psychiatry Psychiatr Epidemiol. 2016;51(3):465–74.
41. Ali A, Hassiotis A, Strydom A, King M. Self stigma in people with intellectual disabilities and courtesy stigma in family carers: A systematic review. Res Dev Disabil [Internet]. 2012;33(6):2122–40. Available from: http://dx.doi.org/10.1016/j.ridd.2012.06.013
42. Ali A, Scior K, Ratti V, Strydom A, King M, Hassiotis A. Discrimination and Other Barriers to Accessing Health Care: Perspectives of Patients with Mild and Moderate Intellectual Disability and Their Carers. PLoS One. 2013;8(8).
43. Thygesen JH, Wolfe K, McQuillin A, Viñas-Jornet M, Baena N, Brison N, et al. Neurodevelopmental risk copy number variants in adults with intellectual disabilities and comorbid psychiatric disorders. Br J Psychiatry. 2018;212(5):287–94.
44. Wolfe K, McQuillin A, Alesi V, Boudry Labis E, Cutajar P, Dallapiccola B, et al. Delineating the psychiatric and behavioral phenotype of recurrent 2q13 deletions and duplications. Am J Med Genet Part B Neuropsychiatr Genet. 2018;177(4):397–405.
45. Wolfe K, Strydom A, Morrogh D, Carter J, Cutajar P, Eyeoyibo M, et al. Chromosomal microarray testing in adults with intellectual disability presenting with comorbid psychiatric disorders. Eur J Hum Genet. 2016;25(1):66–72.
46. Kouimtsidis C, Bosco A, Scior K, Baio G, Hunter R, Pezzoni V, et al. A feasibility randomised controlled trial of extended brief intervention for alcohol misuse in adults with mild to moderate intellectual disabilities living in the community; The EBI-LD study. Trials. 2017;18(1):1–12.
47. Kouimtsidis C, Scior K, Baio G, Hunter R, Pezzoni V, Hassiotis A. Development and evaluation of a manual for extended brief intervention for alcohol misuse for adults with mild to moderate intellectual disabilities living in the community: The EBI-LD study manual. J Appl Res Intellect Disabil. 2017;30(July):42–8.
48. Kouimtsidis C, Fodor-Wynne L, Scior K, Hunter R, Baio G, Pezzoni V, et al. Extended brief intervention to address alcohol misuse in people with mild to moderate intellectual disabilities living in the community (EBI-ID): Study protocol for a randomised controlled trial. Trials. 2015;16(1):1–8.
49. Illingworth JL, Watson P, Ring H. Why do seizures occur when they do? Situations perceived to be associated with increased or decreased seizure likelihood in people with epilepsy and intellectual disability. Epilepsy Behav [Internet]. 2014;39:78–84. Available from: http://dx.doi.org/10.1016/j.yebeh.2014.08.016
50. Illingworth JL, Watson P, Xu S, Manford M, Ring H. A method for identifying associations between seizures and possible trigger events in adults with intellectual disability. Epilepsia. 2015;56(11):1812–8.
51. Maras A, Schroder CM, Malow BA, Findling RL, Breddy J, Nir T, et al. Long-term efficacy and safety of pediatric prolonged-release melatonin for insomnia in children with autism spectrum disorder. J Child Adolesc Psychopharmacol. 2018;28(10):699–710.
52. Gringras P, Nir T, Breddy J, Frydman-Marom A, Findling RL. Efficacy and Safety of Pediatric Prolonged-Release Melatonin for Insomnia in Children With Autism Spectrum Disorder. J Am Acad Child Adolesc Psychiatry [Internet]. 2017;56(11):948-957.e4. Available from: https://doi.org/10.1016/j.jaac.2017.09.414
53. Schroder CM, Malow BA, Maras A, Melmed RD, Findling RL, Breddy J, et al. Pediatric Prolonged-Release Melatonin for Sleep in Children with Autism Spectrum Disorder: Impact on Child Behavior and Caregiver’s Quality of Life. J Autism Dev Disord [Internet]. 2019;49(8):3218–30. Available from: https://doi.org/10.1007/s10803-019-04046-5
54. Malow BA, Findling RL, Schroder CM, Maras A, Breddy J, Nir T, et al. Sleep, Growth, and Puberty After 2 Years of Prolonged-Release Melatonin in Children With Autism Spectrum Disorder. J Am Acad Child Adolesc Psychiatry. 2021;60(2):252-261.e3.
55. Hassiotis A, Poppe M, Strydom A, Vickerstaff V, Hall IS, Crabtree J, et al. Clinical outcomes of staff training in positive behaviour support to reduce challenging behaviour in adults with intellectual disability: Cluster randomised controlled trial. Br J Psychiatry. 2018;212(3):161–8.
56. Ali A, Blickwedel J, Hassiotis A. Interventions for challenging behaviour in intellectual disability. Adv Psychiatr Treat. 2014;20(3):184–92.
57. Ratti V, Hassiotis A, Crabtree J, Deb S, Gallagher P, Unwin G. The effectiveness of person-centred planning for people with intellectual disabilities: A systematic review. Res Dev Disabil. 2016;57(Id):63–84.
58. Ratti, V. *et al.* (2017) ‘An Exploratory Factor Analysis and Construct Validity of the Resident Choice Assessment Scale With Paid Carers of Adults With Intellectual Disabilities and Challenging Behavior in Community Settings’, *Journal of Mental Health Research in Intellectual Disabilities*, 10(3), pp. 198–216. doi: 10.1080/19315864.2016.1277287.
59. Melville CA, Mitchell F, Stalker K, Matthews L, McConnachie A, Murray HM, et al. Effectiveness of a walking programme to support adults with intellectual disabilities to increase physical activity: Walk well cluster-randomised controlled trial. Int J Behav Nutr Phys Act [Internet]. 2015;12(1):1–11. Available from: http://dx.doi.org/10.1186/s12966-015-0290-5
60. Mitchell F, Stalker K, Matthews L, Mutrie N, Melling C, McConnachie A, et al. A qualitative exploration of participants’ experiences of taking part in a walking programme: Perceived benefits, barriers, choices and use of intervention resources. J Appl Res Intellect Disabil. 2018;31(November 2016):110–21.
61. Melville CA, Johnson PCD, Smiley E, Simpson N, Purves D, McConnachie A, et al. Problem behaviours and symptom dimensions of psychiatric disorders in adults with intellectual disabilities: An exploratory and confirmatory factor analysis. Res Dev Disabil. 2016;55(April):1–13.
62. Melville CA, Johnson PCD, Smiley E, Simpson N, McConnachie A, Purves D, et al. Statistical modelling studies examining the dimensional structure of psychopathology experienced by adults with intellectual disabilities: Systematic review. Res Dev Disabil. 2016;53–54(February):1–10.
63. Tuffrey-Wijne I, Goulding L, Gordon V, Abraham E, Giatras N, Edwards C, et al. The challenges in monitoring and preventing patient safety incidents for people with intellectual disabilities in NHS acute hospitals: evidence from a mixed-methods study. BMC Health Serv Res [Internet]. 2014;14:432. Available from: http://ovidsp.ovid.com/ovidweb.cgi?T=JS&PAGE=reference&D=emed15&NEWS=N&AN=609263817
64. Tuffrey-Wijne I, Goulding L, Giatras N, Abraham E, Gillard S, White S, et al. The barriers to and enablers of providing reasonably adjusted health services to people with intellectual disabilities in acute hospitals: Evidence from a mixed-methods study. BMJ Open. 2014;4(4):1–10.
65. Tuffrey-Wijne I, Abraham E, Goulding L, Giatras N, Edwards C, Gillard S, et al. Role confusion as a barrier to effective carer involvement for people with intellectual disabilities in acute hospitals: findings from a mixed-method study. J Adv Nurs. 2016;72(11):2907–22.
